# Design, development, and validation of a multimodal synergy-based intuitive virtual and augmented reality therapy platform for mental health

**DOI:** 10.3389/frobt.2026.1767798

**Published:** 2026-05-04

**Authors:** Parthan Olikkal, Oritsejolomisan Mebaghanje, Viraj Janeja, Sruthi Sundharram, Golnaz Moharrer, Akshara Ajendla, Andrea Kleinsmith, Ann Sofie Clemmensen, Rajasekhar Anguluri, Adam Culbreth, Ramana Vinjamuri

**Affiliations:** 1 Vinjmauri Lab, Department of Computer Science and Electrical Engineering, University of Maryland Baltimore County, Baltimore, MD, United States; 2 Affective Behavior Interaction Lab, Department of Information Systems, University of Maryland Baltimore County, Baltimore, MD, United States; 3 Department of Dance, University of Maryland Baltimore County, Baltimore, MD, United States; 4 Department of Psychiatry, School of Medicine, University of Maryland, Baltimore, MD, United States

**Keywords:** affective computing, dance, human-robot interaction, mental health therapy, multimodal sensing, real-time feedback, synergies, virtual reality

## Abstract

Embodied therapies such as movement therapy have shown promise in enhancing emotional regulation, cognitive engagement, and physical rehabilitation. However, scalable and personalized delivery of such interventions remains a critical challenge. This work presents SIVAM (Synergy-based Intuitive Virtual and Augmented Mental Health platform), a multimodal system that integrates immersive virtual environments, markerless motion capture, physiological sensing, and humanoid robotic mirroring to support affect-aware interventions for mental health. SIVAM combines RGB camera-based skeletal tracking with EEG, EMG, ECG, GSR, and skin temperature sensing using a wearable dry electrode headset to create a closed-loop therapeutic framework. Movement synergies–low-dimensional coordinated patterns across body joints and muscles–are extracted from motion data and aligned with physiological signals to infer affective and motor states in real time, serving as potential biomarkers of stress. The system further introduces a plane-wise movement model that enables natural 3D avatar navigation using a single RGB camera, enhancing embodiment and interaction with virtual environments. A pilot study (N = 5) with five participants of varying dance experience demonstrated reliable motion tracking, real-time synchronization of physiological and movement data, and robust avatar and robot mirroring across diverse movements. These results highlight the feasibility of combining multimodal sensing, virtual avatars, and socially assistive robots to enable scalable, home-based movement therapy.

## 1 Introduction

### 1.1 Background and motivation

Population aging presents a critical public health challenge, particularly in addressing undertreated mental health conditions that impair quality of life. Approximately 25% of adults in the U.S. are affected annually, and mental health disorders globally account for one-sixth of all years lived with disability, yet these conditions are often met with delayed or inaccessible treatment due to stigma, financial burdens, and insufficient specialized care infrastructure (Mental Health, 2026; Mental Health Statistics, 2025; Vos et al., 2020; Reinert and Fritze, 2024). Traditional mental health interventions experience an average delay of up to 11 years from onset, exacerbating symptoms and impacting wellbeing (Wang et al., 2005).

Dance-based movement therapies (DMTs) offer a promising non-pharmacological alternative incorporating physical, sensory, cognitive, and social engagement. A meta-analysis of 14 randomized controlled trials (RCTs) involving 983 older adults affirmed DMT efficiency in enhancing psychological wellbeing and cognitive functioning (Podolski et al., 2023). As highlighted in (Goodill, 2005; Quiroga Murcia et al., 2010; Karkou et al., 2019; Koch et al., 2019) DMTs can alleviate symptoms of stress, anxiety, and depression, while fostering mood enhancement, proprioceptive awareness, and social connectedness. Additional studies demonstrate effectiveness in reducing neuropsychiatric symptoms in dementia patients (Tao et al., 2023), improving quality of life in Parkinson’s disease (Westheimer et al., 2015), and contributing to neuroprotective mechanisms in older adults at risk for Alzheimer’s disease (Kattenstroth et al., 2013; Rehfeld et al., 2018). Beyond mental health, DMT also supports motor and cognitive resilience by improving postural control, sensorimotor coordination, and executive functioning (Rehfeld et al., 2018; Menezes et al., 2022; Podolski et al., 2023).

Central to DMT’s therapeutic efficacy is its reliance on coordinated interlimb movement and dynamic muscle synergy recruitment. These synergies reflect neuromotor strategies underlying functional gestures and serve as sensitive biomarkers of both emotional and neurological states (Ting and McKay, 2007; d’Avella and Lacquaniti, 2013). From an affective neuroscience standpoint, techniques such as multivariate pattern analysis (MVPA) enable real-time decoding of affective states from neural signals, offering mechanistic insights into the neuromodulatory impact of dance therapy (Kragel and LaBar, 2016). Although Podolski et al. (2023) note that most reviewed RCTs lacked direct neuroplasticity measures, converging evidence suggests the DMT’s capacity to influence brain function through movement. This aligns with a growing body of evidence demonstrating neuroadaptive changes–such as enhanced functional connectivity within emotion-and cognition-related brain networks (e.g., default mode and fronto-parietal networks) and improved white matter integrity in tracts like the fornix–supporting long-term cognitive-emotional resilience in aging individuals, particularly those with subjective cognitive decline (SCD) or mild cognitive impairment (MCI) (Burzynska et al., 2017; Teixeira-Machado et al., 2019; Balazova et al., 2021).

Despite the compelling therapeutic potential of DMT, broader deployment continues to face significant structural and practical constraints. Traditional in-person sessions are inherently resource-intensive, requiring trained therapists, dedicated spaces and accessible transportation, making them less accessible to individuals with mobility limitations or those residing in rural areas. As a result, consistent participation and long-term adherence are frequently compromised, thereby limiting the full therapeutic impact of DMT interventions.

### 1.2 Related work

Dance movement integration, including formal DMT, has consistently shown positive effects on mental, emotional, and cognitive health in diverse populations. Recent efforts have moved these interventions into technology-mediated contexts. Radanliev (2024) emphasizes the necessity of embodied experience, wearable feedback, and ethical design in XR-based therapeutic systems. In a follow-up study, Radanliev (2025) implemented a Virtual Dance Movement Therapy (XR-DMT) framework that uses biometric signals such as electroencephalography (EEG), electrodermal activity (EDA), heart rate variability (HRV), skin conductance and AI to tailor dance sessions for anxiety reduction. However, these systems lack integration of robotic agents or interactive avatars with synchronized motion and physiological feedback.

A key principle for seamless translation of complex human motion into its fundamental components is motor synergies–coordinated patterns of muscle or joint activation that represents a fundamental strategy of the nervous system for simplifying control of complex movements. Merel et al. (2019) proposed a neural probabilistic motor primitive framework for full-body humanoid control, enabling one-shot imitation and generalization across novel tasks. Comparisons between kinematic primitives and mechanical impedance primitives highlight the value of combining low-dimensional movement encoding with dynamic compliance to improve robustness and naturalistic behavior in interactive robotic systems (Nah et al., 2024). Olikkal et al. (2023) introduced a MediaPipe-based gesture recognition framework that extracts 33 hand landmarks and encodes them into low-dimensional kinematic synergies (Olikkal et al., 2022), which preserved over 98% of original movement variance. These synergies were then successfully deployed to reconstruct gestures with high fidelity on a humanoid robot. Building on this framework, Olikkal et al. (2024a) demonstrated real-time robotic control using synergy-based mapping of human arm gestures, offering a compact yet expressive representation of movement. Malhotra et al. (2025) introduced SynSculptor, a postural synergy-based scripting framework that uses PCA-extracted human synergies to generate stylized robotic movements without retraining.

Robot-facilitated movement therapy has shown promise in enhancing engagement and supporting physical outcomes. Bevilacqua et al. (2025) combined Irish dance with robotic guidance for Parkinson’s therapy, resulting in improvements in balance and gait, though it lacked affective or avatar feedback. Li et al. (2022) conducted a pilot study in long-term care, finding that older adults responded positively to robot-facilitated dance sessions. Chen et al. (2011) explored partner-dance with a mobile manipulator, advancing embodied interaction. Javed and Park (2022) investigated real-time adaptation and leader-follower dynamics for engaging vulnerable populations. While each of these systems addresses facets of physical or social robots, few unify movement, emotion, and avatar/robot feedback in a single platform.

Motion-based emotion classification has received increasing attention. Karumuri et al. (2019), applied convolutional neural networks to MoCap dance data to classify emotional states such as anger, happiness, sadness, insecurity, achieving F1 scores up to 0.79. Bai et al. (2021) proposed a hybrid feature set for emotional classification in dance movements. Foundational work by Camurri et al. (2003) examined expressive gesture recognition from full-body dance, establishing core methods for automated emotion inference from movement. Piana et al. (2014) achieved approximately 61.3% accuracy in real-time emotion recognition from body gestures. Turab et al. (2025) recently introduced Laban Movement Analysis (LMA) feature descriptors for 3D key point motion data and achieved as high as 96.85% classification accuracy.

Emotional awareness and regulation are pivotal for cognitive wellbeing, stress resilience, and performance enhancement (Sutton, 2016; Ferguson and Lockman, 2024), with poor regulation depleting psychological and social resources in high-stress environments (Akiri et al., 2024). Dance and movement-based practices have been used to improve mood and reduce rumination (Mackie and Bates, 2019; Ajendla et al., 2025; Olikkal et al., 2026), though primarily within expressive or therapeutic populations (Mateu et al., 2021). To broaden applicability, recent works recommend embedding tangible and reflective technologies that support embodied emotional practice (Hollis et al., 2017; Daudén Roquet et al., 2022). Advances in affective computing demonstrate that physiological signals such as EEG, electrocardiogram (ECG) and galvanic skin responses (GSR) can detect emotional states with accuracy reaching up to 81% for extreme arousal or valence levels (Horlings et al., 2008). These findings underscore the potential for integrating emotion-aware, body-based, interactive systems as tools to foster emotional resilience and mental wellbeing.

### 1.3 Research gap and key contributions

Despite major advancements in DMT, affective computing, and robot-assisted interventions, existing systems continue to face several critical limitations that constrain their therapeutic impact and accessibility. As summarized in Table 1, most platforms lack real-time integration of physiological signals with full-body motion tracking, limiting their capacity to deliver synchronized and adaptive emotional feedback. Additionally, many systems do not include emotionally responsive avatars or real-time social feedback mechanisms, such as partner-based or multiplayer interaction–omissions that significantly reduce the depth of therapeutic engagement and personalization. Accessibility further remains a major barrier, as many platforms rely on clinic-based equipment, technical supervision, or high-bandwidth environments, rendering them impractical for widespread deployment among older adults, home-bound individuals, or those in underserved communities.

**TABLE 1:** Comparison summary of SIVAM platform across six dimensions.

System	Data capture methods	Physiological sensing	Robot integration	Avatar feedback	Synergy-based	Real-time
Radanliev (2024), Radanliev (2025)	XR-based dance session with embodied interaction	EEG, EDA, HRV, skin conductance; wearable feedback	No	Yes	No	Yes
Merel et al. (2019)	Expert motion demonstration	No	Yes	No	Yes	Yes
Nah et al. (2024)	Conceptual comparison of kinematic and impedance primitives	No	Yes	No	Yes	Not specified
Olikkal et al. (2022), Olikkal et al. (2023), Olikkal et al. (2024a)	MediaPipe-based hand and arm landmark extraction	No	Yes	No	Yes	Yes
Malhotra et al. (2025)	PCA-extracted human posture monitoring	Not mentioned	Yes	No	Yes	Not specified
Bevilacqua et al. (2025)	Dance with robotic posture monitoring	Not mentioned	Yes	No	No	Not specified
Li et al. (2022)	Robot-facilitated dance sessions	Not mentioned	Yes	No	No	Not specified
Chen et al. (2011)	Partner-dance with mobile manipulator	Not mentioned	Yes	No	No	Not specified
Javed and Park (2022)	Leader-follower dance dynamics	Not mentioned	Yes	No	No	Yes
SIVAM (ours)	RGB	EEG, ECG, GSR, EMG, temp	Yes (mitra, G1)	Yes (dual avatar)	Yes	Yes

**FIGURE 1:**
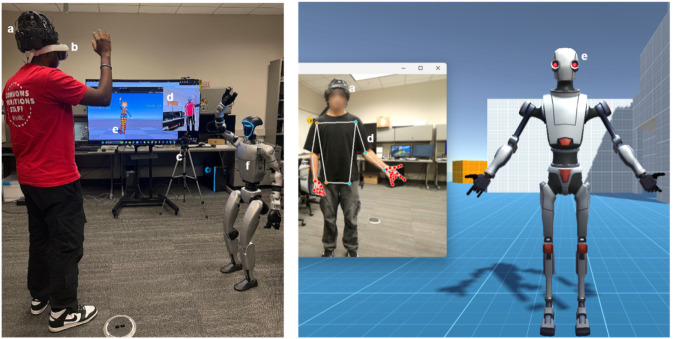
Overview of the SIVAM platform illustrating real-time multimodal interaction for mental health support is shown here. The system integrates **(a)** a dry-electrode EEG system for non-invasive brain signal acquisition, **(b)** a head-mounted display (HMD) providing immersive visual feedback, **(c)** an RGB-based movement tracking camera for capturing full-body motion, **(d)** a custom joint-mapping framework for translating user kinematics to digital and robotic avatars, **(e)** an animated avatars that interacts with the user in virtual space, **(f)** a humanoid robot partner that replicates upper-limb gestures to support embodied, affective engagement in therapeutic dance interaction.

To address these challenges, this paper introduces SIVAM (Synergy-based Intuitive Virtual and Augmented Therapy for Mental Health), a technology-enabled platform designed for personalized, home-based therapy that integrates DMT principles with humanoid robotics to offer non-stigmatizing, socially engaging therapeutic sessions within the user’s own environment (Figure 1). Leveraging social assistive robots that provide non-judgmental companionship and motivation (Tapus et al., 2007), SIVAM replicates the social dynamics of group-based DMT while eliminating logistical barriers such as transportation or physical accessibility.

A key feature of SIVAM is its use of interlimb coordination patterns as interpretable indicators of motor and affective state during movement interaction. Through markerless motion capture and neurophysiological monitoring, the system enables real-time analysis of movement and affective states during embodied interaction. This facilitates comparative studies between human and robot dance partners to assess therapeutic outcomes and optimize system behavior.

The specific research questions addressed are:

- Can a single RGB camera system provide reliable real-time motion capture for therapeutic applications across varying distances and movement complexities?
- Can physiological modalities such as EEG, EMG, ECG, GSR, skin temperature be synchronized with movement data to enable synergy-based affective state inference?
- Does the platform demonstrate sufficient robustness in tracking accuracy, real-time responsiveness, and user engagement to support future clinical validation with target population?

As summarized in Table 1, SIVAM integrates single RGB markerless motion capture, plane-wise avatar navigation, multimodal physiological sensing through a wearable dry-electrode headset, humanoid robot mirroring, and synergy-based motor encoding within an immersive VR environment.

This work reports the system design and initial technical validation with five healthy participants, establishing feasibility prior to clinical trials. The key contributions are:

- A novel architecture synchronizing five physiological modalities with RGB-based motion capture and mapping to virtual avatars and humanoid robots in real-time
- Two distinct interaction modes–mirroring for self-observation and learning for structured imitation–that preserve therapeutic principles while enabling asynchronous home practice
- Initial validation with participants across different dance skill levels demonstrating system robustness, tracking fidelity, and physiological synchronization sufficient for progression to target populations.

**FIGURE 2:**
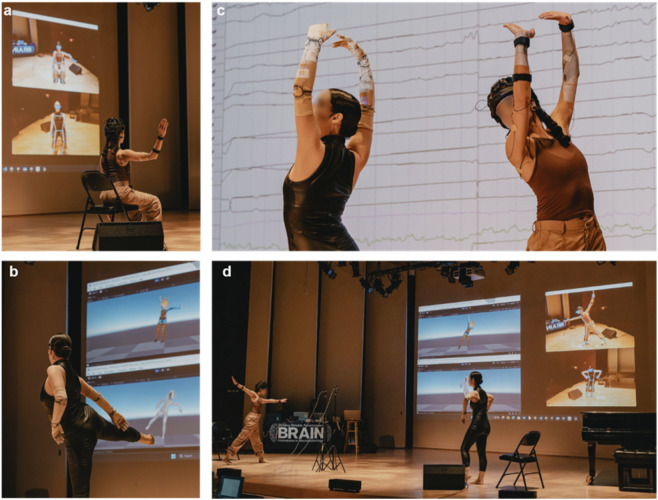
Expert dancers interacting with the SIVAM platform during multimodal dance-based sessions. **(a)** Physiological signals, including EEG, heart rate variability, and skin temperature, are recorded using a dry-electrode system to assess cognitive and affective states in real-time. **(b)** A swivel-mounted RGB camera captures full-body landmarks via 2D pose estimation, enabling real-time mapping of skeletal movements onto animated avatars in the Unity environment. **(c)** Synchronized visualizations of physiological signals (background) offer insights to the dancers’ mental and emotional engagement during the session. **(d)** Dual-user interaction showcasing the SIVAM platform’s multi-user capability, where two participants engage simultaneously with full-body tracking and avatar rendering.

SIVAM’s interdisciplinary framework blends affective neuroscience, expressive motor coordination, and biologically inspired robotics into a cohesive therapeutic experience. This design supports emotional wellbeing, neuromotor rehabilitation and aging-in-place strategies for older adults. It directly answers the call by Podolski et al. (2023) for accessible and equitable mental healthcare solutions and supports the broader integration of DMT into healthcare ecosystems as advocated by Goodill (2005) as shown in Figure 2. For population at risk of cognitive decline, such as individuals with subjective cognitive decline or mild cognitive impairment, SIVAM offers multimodal engagement across physical, psychological, and social dimensions. This multisensory stimulation fosters resilience against neurodegeneration. By tailoring interventions to individual need through robotic adaptation, SIVAM also bridges critical gaps in access for those in rural settings, with mobility constraints, or limited access to specialized care. A limitation of this study is the healthy participant cohort, which was intentional for initial technical validation.

## 2 Methods

### 2.1 Study design and participants

This study was conducted at the University of Maryland Baltimore County’s Vinjamuri Lab between February and July 2025. A technical feasibility design was employed to validate the SIVAM platform before clinical deployment, with objectives to assess tracking fidelity, multimodal synchronization, mirroring accuracy, and usability across user skill levels as outlined in Section 1.3. The study comprised three phases: system development, data collection, and offline performance analysis. The initial participant study was designed as a technical validation experiment to evaluate system robustness, multimodal synchronization, and interaction fidelity, rather than to assess therapeutic outcomes.

Five participants were recruited by members of the Vinjamuri Lab at the University of Maryland Baltimore County through word-of-mouth announcement. Recruitment and eligibility screening were conducted by the research team under Institutional Review Board (IRB), approved protocols and all procedures were conducted in accordance with the Declaration of Helsinki and participants provided written consent. The cohort included two females and three males (22.6 ± 3.8 years). Inclusion criteria were age 18–35 years, ability to perform light physical activity, and no diagnosed mental health condition or mobility impairments. Exclusion criteria included history of seizures, medications affecting motor function, or inability to provide consent. The cohort comprised three individuals with no formal dance training and two experienced dancers with more than 5 years of training, intentionally selected to stress-test the system across different motion complexities.

**FIGURE 3:**
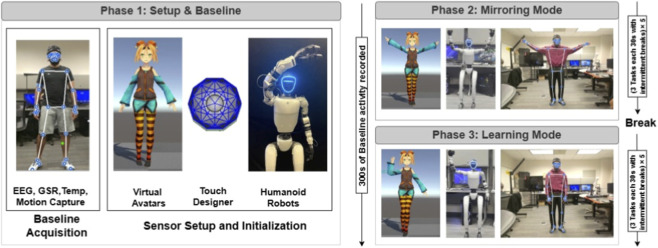
Experimental workflow of SIVAM. The study consists of three phases: (1) Setup and baseline Acquisition, where multimodal physiological signals (EEG, GSR, skin temperature) and motion tracking data are initialized; (2) Mirroring mode, in which participants perform movements that are replicated in real time by a virtual avatar or humanoid robot; and (3) Learning mode, where the avatar or robot demonstrates predefined movements for the participant to imitate. Each of the mode has three tasks which each task having a time constraint of 30s. These tasks were repeated five times. Adequate breaks were provided between the tasks and between the modes.

At the beginning of each experimental session, participants were seated comfortably in a chair while the wearable sensing equipment was prepared. A dry-electrode EEG headset (DSI-24, Wearable Sensing) was first mounted on the participant’s head, and electrode impedance levels were monitored using the manufacturer’s acquisition software to ensure optimal signal quality as shown in Figure 3. The system interface provided real-time feedback on electrode contact, allowing adjustments until acceptable impedance levels were achieved. Once the EEG signals were stabilized, additional physiological sensors were attached according to the recommended sensor placements. After sensor preparation, participants were asked to stand to perform the movement-based interaction tasks. To prevent interference with hand movements during the gesture and dance sequences, sensor cables were secured using medical tape along the participant’s arms to minimize cable motion and avoid tangling during movement. Following sensor preparation, the camera-based motion capture system was initialized and to detect the participant’s body landmarks.

### 2.2 System architecture

**FIGURE 4:**
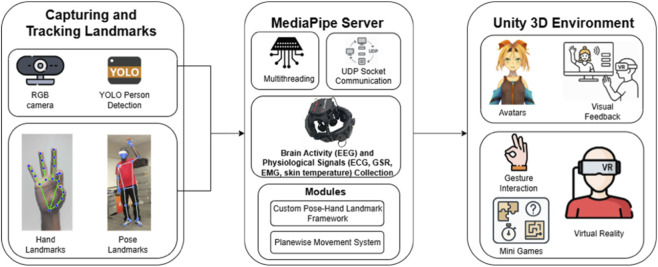
System architecture of the SIVAM platform. The core components include a MediaPipe server that detects full-body and hand landmarks from an RGB camera feed, with these 2D landmarks transmitted via UDP socket communication to the Unity3D environment. In Unity, the landmarks are mapped in real time onto custom virtual avatars, enabling gesture-based interaction, biofeedback, and immersive mini-games for therapeutic engagement. The system supports both desktop and VR modes to enhance embodiment and user immersion. In parallel, multimodal physiological signals–including EEG, ECG, GSR, EMG, and skin temperature–are acquired and time synchronized, allowing closed-loop mapping between movement execution and emotional or cognitive states. These data streams also support synergy extraction that can act as biomarkers for stress detection.

SIVAM is structured into two primary modules that operate simultaneously: (i) a Python-based MediaPipe Server and (ii) a Unity 3D environment. The architectural diagram of SIVAM platform is shown in Figure 4. The MediaPipe Pose landmark model (Bazarevsky et al., 2020) captures 33 full-body skeletal landmarks whereas the MediaPipe Hand landmark model (Zhang et al., 2020) captures 21 landmarks per hand through a single RGB camera. These streams are fused into a custom 67-point landmark representation. To ensure smooth data flow and minimal latency, data transfer between the backend and Unity environment occurs via UDP sockets with multithreading to ensure non-blocking processing of the pose pipeline and communication. The Unity environment handles real-time avatar animation, user feedback, gameplay interaction, and virtual reality (VR) integration. Landmark data are received and mapped to avatar joints using custom C# scripts, which employs inverse kinematics and smoothing interpolation to simulate natural motion.

### 2.3 Landmark capture, avatar mapping, and plane-wise motion integration

The SIVAM platform captures full-body and fine-grained hand movements through MediaPipe Pose, MediaPipe Hand, and a custom landmark fusion framework. This framework enables concurrent tracking of gross motor behaviors (e.g., arm and leg movements) and fine motor gestures (e.g., finger articulation), producing a unified 67-point landmark representation for high-resolution motion analysis. To extend beyond single-user scenarios, a YOLO-based detection module identifies multiple individuals within the camera frame, although only one user currently controls the avatar.

The extracted landmarks are mapped in real time to a 3D avatar within the Unity environment, providing visual feedback that mirrors the user’s movements. SIVAM supports two avatars optimized for distinct therapeutic contexts: FlightUnit (2020) for full-body tracking and immersive VR sessions, and Brewer (2026) for high-fidelity hand tracking and gesture-specific rehabilitation. Unity-Chan supports expressive full-body motion suitable for dance-based interaction, whereas Kyle Robot focuses on accurate hand-pose rendering for fine-motor rehabilitation and gesture-based emotional expression.

This dual avatar architecture allows SIVAM to adapt to different therapy tasks, ensuring that both large-scale body movements and detailed hand gestures can be represented accurately. Landmark coordinates extracted from the RGB camera are transmitted to Unity environment and mapped to avatar joints, enabling responsive visual feedback for complex movements such as sign-language gestures, intricate hand-based choreographies, or complex full-body dance sequences.

A notable innovation in SIVAM is the plane-wise movement system, which allows users to move fluidly in three dimensions–left-right (x), up-down (y), and forward-backward (z) – relative to their position and scale within the camera frame. This mechanism allows for continuous avatar locomotion within the Unity 3D environment without requiring external controllers or trackers. This spatial mapping enhances embodiment, presence, and agency, thereby deepening the therapeutic potential of immersive environments.

While prior systems (Strutt and Cisneros, 2021; Weber and Cook, 2022) have demonstrated avatar-based dance co-creation and embodied XR performance, neither implements continuous depth-based avatar navigation or real-time translational control. These platforms typically focus on mirroring, gestural expression, or perceptual adaptations, with limited avatar mobility confined to a static or predefined location. In contrast, SIVAM’s plane-wise movement system not only tracks fine and gross motor gestures, but also dynamically maps the user’s position in 3D space to the avatar’s locomotion. This engages users in a naturalistic spatial interaction, such as approaching a virtual therapist, moving toward a target location or participating in choregraphed spatial routines.

### 2.4 Clinical workflow and interactive module

The SIVAM platform is designed to extend access to structured, embodied therapeutic interaction rather than replace therapists. The system enables users who cannot regularly attend in-person sessions to engage in guided movement-based interaction through virtual avatars and humanoid robots. To support different therapeutic goals, SIVAM provides two interaction modalities: (a) mirroring mode, and (b) learning mode.

- Mirroring mode: In this mode, the avatar or robot replicates the user’s movements in real-time from a first-person perspective. When the user raises their right arm, the avatar’s right arm rises correspondingly. This mode is designed for self-observation and motor awareness, allowing the user to see their own movement and adjust their posture or expression. It is analogous to practicing in front of a mirror. Thus, when the robot faces the user, the correspondence is specular, that is the user’s right to robot’s left, to preserve the experience of looking into a mirror.
- Learning mode: In this mode, the avatar or robot acts as a teacher, performing a pre-recorded or therapist-demonstrated gesture sequence. The user is promoted to imitate the movement within a time window. This mode supports structured motor learning, cognitive sequencing and goal directed engagement. It is analogous to therapist saying, “Watch me, now you try”. Here, when the robot faces the same direction as the user, the correspondence is laterality, that is user’s right to robot’s right.

**FIGURE 5:**
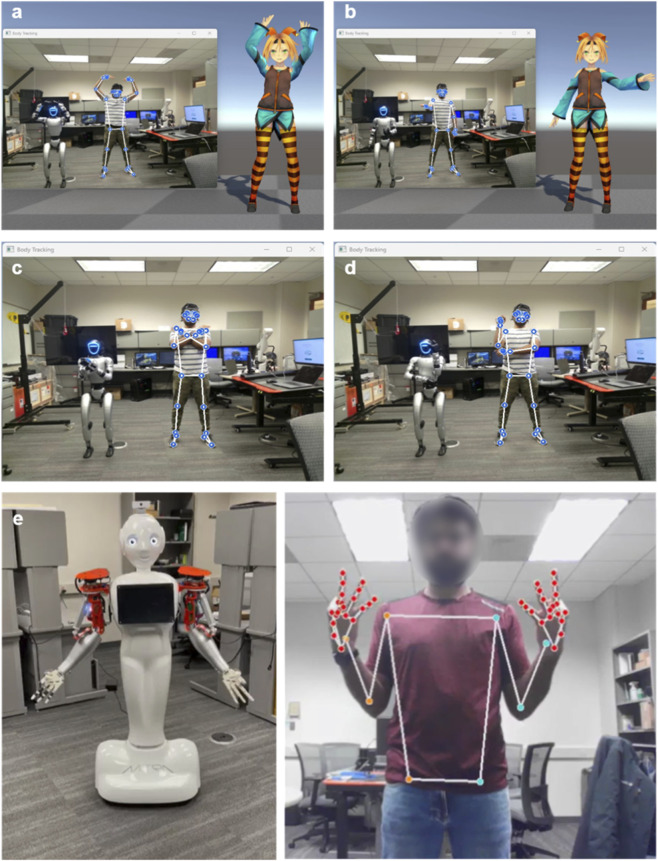
This figure illustrates the integration of landmark models with humanoid robots for real-time movement mirroring. The **(a–e)** demonstrate the SIVAM platform’s capability to map human movement onto robotic agents using pose landmarks extracted from an RGB camera stream. **(a,b)** show the user performing movements simultaneously mirrored by virtual avatar and the G1 humanoid robot. In this mode, the robot’s left hand corresponds to the user’s right hand, preserving the experience of mirror reflection. **(c,d)** depicts the G1 robot replicating the user’s movements independently. **(e)** illustrates the custom pose-hand landmark mapped to Mitra robot which features articulated fingers for replicating fine-grained gestures. The user’s movements are captured via RGB camera and processed through MediaPipe’ pose and hand landmarks which were mapped to G1 and Mitra in real-time, enabling embodied human-robot interaction.

These interaction modes replicate key elements of therapist-guided DMT, including observation, imitation, and feedback-based motor learning. As illustrated in Figure 5, these mappings allow the system to support both self-observation and guided imitation depending on therapeutic needs. Therapists can record gesture demonstrations or short dance sequences that are stored in a session library. These therapist-authored sequences can later be accessed by patients during independent sessions. While interacting using the system, users receive real-time feedback on movement accuracy, and both physiological and kinematic data are logged for asynchronously review by the therapist to monitor progress and adjust the intervention.

To support complex movement training, SIVAM incorporates a sequential choreography playback mechanism, allowing multiple gestures to be demonstrated as coordinated movement sequences. During playback, the avatar performs a predefined movement sequence, after which the user attempts to reproduce the same choreography. The system evaluates performance by comparing user joint trajectories with reference motion, enabling feedback on timing and movement similarity. A central feature of the platform is its interactive gesture-matching mini-game, designed to improve coordination and engagement. In each round, the avatar demonstrates a target gesture and the user attempts to replicate it within a fixed time window. Gesture recognition is performed by evaluating joint relationships derived from MediaPipe landmarks streamed to Unity. For example, a “Raise Left Arm” is detected when the left wrist exceeds the shoulder height, while a “Hands on Hips” pose is identified through spatial proximity between wrist and hip landmarks. Successful execution triggers positive visual feedback, while incorrect attempts prompt corrective cues. To support both training and rehabilitation, SIVAM also enables recording and playback of user-performed gestures. This functionality allows previously executed movements to be replayed and compared with reference gestures, enabling repeated practice and tracking of motor performance.

Earlier works (Chan et al., 2011) introduced a virtual teacher avatar-based system for mirroring dance movements and feedback in VR using motion capture. By wearing bodysuits for motion tracking, users’ motions were captured, analyzed and feedback were provided through the avatar. While previous systems such as Cha et al. (2021) introduced real-time upper-limb and finger avatar tracking for virtual rehabilitation, their focus remained on task-based motion in VR, lacking the sequential gesture chaining and mirrored playback that SIVAM offers. Similarly, Khan et al. (2020) designed a rhythmic stepping-based avatar interaction model to aid gait training, incorporating audio-visual cues but not gesture-level tracking or gesture playback logic, and with no fine motor expression via hand pose recognition.

### 2.5 Virtual reality integration for embodied therapy

To enhance the immersive experience and address challenges in readability and engagement–especially during fast-paced gesture-matching tasks–SIVAM is fully compatible with VR headsets, including the Meta Quest 2 and Meta Quest 3. This integration enables users to interact with the system in a deeply embodied and spatially dynamic manner, fostering stronger emotional connection, attention, and therapeutic presence. In VR mode, users have the option to engage with the platform in either: first-person perspective, where they inhabit their avatar’s point of view and experience full-body movement from an egocentric frame, reinforcing self-agency and ownership of motion, or third-person perspective, where they can observe their avatar performing choreographed sequences and evaluate their own form and timing with greater external awareness. Crucially, all core functionalities–real-time gesture tracking, matching, scoring feedback, and avatar animation–are preserved and synchronized in VR mode without requiring any additional instrumentation. This seamless transition from 2D desktop-based interaction to a 3D virtual environment ensures consistent accessibility across different user needs and therapeutic settings.

### 2.6 Humanoid robots

The SIVAM platform integrates two different humanoid robots (Figure 5), Mitra (Invento Research Inc., Plano, TX) and Unitree G1 EDU U2 (Unitree Robotics Inc., Hangzhou, China).

Mitra is a humanoid robot designed for expressive upper-body interaction, featuring 21 degrees of freedom (DoFs) across head, arms and hands. Each hand comprises 5 DoFs dedicated to individual finger articulation, supplemented by 1 DoF at the wrist, one at the elbow, 2 at each shoulder, one for head motion, and 2 additional DoFs at the mobile base for locomotion. An onboard RGB camera ( 1280×720 resolution) mounted on the head provides visual sensing for environmental awareness, while a chest-mounted iPad (ninth Gen) functions as a user interface. Within the SIVAM framework, Mitra is primarily used for mirroring fine-motor gestures and hand-based movements, supporting expressive and symbolic components of dance-based interaction.

Unitree G1 is a full-body humanoid system with 43 DoFs, including 6 DoFs per leg, 5 DoFs per arm, 3 DoF in the waist, 7 DoFs per hand and 2 DoFs per wrist, enabling dynamic whole-body movement and locomotion. The robot incorporates torque-controlled actuators and integrated sensors that support stable bipedal motion and responsive upper-body gestures. These capabilities make the G1 suitable for mirroring large-scale body movements and dance-based limb coordination.

### 2.7 Multimodal data collection for real-time affective and synergy monitoring

The SIVAM platform integrated multimodal physiological sensing with motion tracking to monitor users’ affective, cognitive, and neuromotor states during therapeutic interaction. Physiological signals are acquired using the DSI-24 dry-electrode EEG headset (Wearable Sensing, San Diego, United States), a wireless, wearable device capable of simultaneously recording EEG, EMG, GSR, ECG and skin temperature. These signals are recorded synchronously during interactive activities such as dance sequence execution and gesture-matching tasks.

Each physiological modality provides complementary information about the user’s internal state. EEG captures neural dynamics related to cognitive engagement and attentional processes (Alarcao and Fonseca, 2019). EMG reflects muscle activation patterns associated with motor effort and gesture execution (Olikkal et al., 2024b). GSR and skin temperature provide indicators of autonomic nervous system activity linked to emotional valence and arousal states (Boucsein, 2012), while ECG data enables real-time heart rate variability (HRV) analysis, a widely used marker of stress regulation, emotional resilience, and relaxation response (Shaffer and Ginsberg, 2017).

No single modality is sufficient for reliable affective state estimation. EEG offers high temporal resolution but poor spatial specificity, whereas GSR is arousal-specific but valence-agnostic. ECG reflects both branches of the autonomic system, and EMG captures motor execution but not autonomic states. Skin temperature changes slowly and cannot capture rapid fluctuations. By combining these complementary physiological signals, SIVAM reduces ambiguity associated with individual modalities and enables a more reliable interpretation of the user’s internal state.

In this work, reliability is the consistent inference of user state through the integration of complementary physiological and behavioral signals, such that limitations or noise in individual modalities do not lead to unstable interpretations. This reliability is evaluated at a system level through signal acquisition, temporal synchronization across modalities, and consistency between physiological indicators and observed movement patterns.

This multimodal sensing enables SIVAM to extract behavioral movement synergies–coordinated low-dimensional motor patterns that emerges across muscles and joints during dance or gesture execution. These synergies can be obtained through dimensionality reduction applied to the landmarks obtained from MediaPipe as detailed in (Olikkal et al., 2023; 2025; Safavi et al., 2024). These synergies serve as interpretable representations of how nervous system organizes movement under different affective states. By comparing these synergy patterns with simultaneously acquired physiological signals (EEG, ECG, GSR, and skin temperature), the platform can infer whether a user is stressed or non-stressed state. This synergy-physiology coupling forms the core analytical mechanism of SIVAM, allowing the system to detect subtle deviations in motor behavior linked to emotional dysregulation and to dynamically adapt therapeutic interventions based on real-time mental state estimation. It is to be noted that this study focuses on technical feasibility, and formal validation of affective state inference against ground-truth labels is part of future work.

#### 2.7.1 Clinical rationale for affective sensing

A dance movement therapist continuously observes not only motor performance but also affective states such as facial expression, posture, muscle tension, and breathing, and implicitly adapts the session in real time (van Geest et al., 2021). This implicit, real-time adaptation is a cornerstone of therapeutic alliance and intervention efficacy. However, in asynchronous or home-based settings, this perceptual channel is lost. The therapist cannot see the patient and the patient may not accurately self-report due to social bias or lack of awareness during movement-focused tasks, and subtle fluctuations in affective states may go unnoticed until they escalate into invisible distress or disengagement.

By continuously estimating sympathetic arousal through GSR and HRV, cognitive load through EEG, and motor indicators of stress through synergy analysis, the SIVAM platform provides a real-time adaptation and generates feedback for the therapist, enabling longitudinal tracking of affective regulation as an outcome metric (Zhang and Wei, 2024). The platform is not aimed at replacing the therapist’s judgement but serves as a sensor-based augmentation of the therapist’s perceptual capabilities when translated to the remote setting.

#### 2.7.2 Deployment considerations

To support practical deployment, SIVAM was designed with several considerations for hardware scalability, usability and energy efficiency.

All physiological signals are acquired via a single, wearable dry-electrode headset (DSI-24 dry EEG), eliminating the need for multiple chest straps, finger electrodes or separate amplifiers. This single device configuration reduces setup time to under 5 minutes, simplifies the sensing architecture, and lowers overall system complexity. The headset samples all signal at 300 Hz using the manufacturer’s acquisition software. It also incorporates several hardware and software features to maintain signal quality during movement including active shielding to reduce electromagnetic interference, real-time impedance monitoring, and spring-loaded dry electrodes that maintain consistent scalp contact. In addition, built-in signal processing includes bandpass filtering (1–50 Hz) and artifact removal for ocular and muscular artifacts from EEG signals help maintain reliable signal acquisition. No user calibration is required prior to each session. A 30 s resting baseline is monitored for all signals. Impedance is monitored continuously, and system indicates user to adjust the headset if contact quality degrades below threshold, visible in the DSI-24 interface.

Pose estimation is performed locally using the MediaPipe framework, achieving a processing rate of more than 30 frames per second on a consumer-grade GPU. The current system is hosted on a workstation equipped with an Intel Core i9-13800 CPU (2.0 GHz) and an NVIDIA GeForce RTX 4070 GPU (12 GB VRAM). Communication between the MediaPipe server and the Unity-based visualization via UDP maintains high data transfer with low computational overhead (less than 10% CPU utilization).

From a sustainability perspective, performing motion tracking and signal processing locally reduces the need for continuous cloud computation and associated network energy costs. The use of a single wearable sensing device further minimizes hardware redundancy and power consumption compared with systems that rely on multiple independent sensors. Although the current prototype operates on a desktop workstation and VR headset, the core architecture of single camera motion tracking, synergy extraction, and closed-loop adaptation has been designed for eventual deployment on a standalone or edge-computing platforms with lower energy requirements.

## 3 Results

### 3.1 Motion capture and avatar mapping performance

Motion data were captured using the Obsbot Tiny 2 AI-powered swivel camera, capable of tracking users at 1920 × 1080 resolution and 60 fps. This ensured reliable capture even as users moved dynamically through physical space. MediaPipe Pose and Hand models were overlaid onto the live RGB feed to extract 33 full body and 21 hand landmarks per hand, which were streamed via UDP to the Unity engine for real-time mapping to avatars. Depending on the therapeutic mode, participants interacted with either the Unity-Chan avatar (for full-body dance sequences) or the Kyle Robot avatar (for fine-motor hand gestures).

To accommodate user skill levels, the system offered two task complexity levels. Non-dancers engaged in rhythmic, low-complexity dance combos and gesture-matching mini-games designed to foster coordination and affective engagement without requiring choreographic expertise. Trained dancers performed expressive (as shown in S1 and S2), high-mobility sequences utilizing the plane-wise movement system, challenging the system’s tracking fidelity, motion mapping, and real-time feedback under more demanding conditions. Demonstrations of the system functionality, including real-time motion tracking, avatar mirroring, and humanoid robot interaction, are provided in the supplementary videos (Supplementary Videos 1, 2).

### 3.2 Physiological signal acquisition and bio-responsive visualization

In addition to motion tracking, multimodal physiological signals were recorded to assess neural and autonomic responses during interaction with the SIVAM platform. Data acquisition was performed using the DSI-24 dry EEG head cap, integrated with additional modules for ECG and skin temperature monitoring. All signals were sampled at 300 Hz, enabling high-resolution analysis of neural and autonomic dynamics. This enabled synchronized analysis of motor execution, affective response, and neural engagement, contributing to the development of personalized feedback algorithms and synergy-based emotional biomarkers.

To explore the relationship between physiological activity and embodied interaction, physiological signals from a representative session were integrated into a bio-responsive visualization environment developed in TouchDesigner. EEG signals were analyzed within the alpha frequency band (8–12 Hz) using fourth-order Butterworth bandpass filter. The analytic envelop of alpha activity was extracted using the Hilbert transform and averaged across channels to generate a global indicator of cortical engagement. ECG signals were processed to derive heart rate dynamics through R-peak detection and computation of beat-to-beat intervals. A moving average filter was applied to smooth the resulting heart rate signal. Skin temperature measurements were temporally synchronized with EEG and ECG signals to enable multimodal analysis.

**FIGURE 6:**
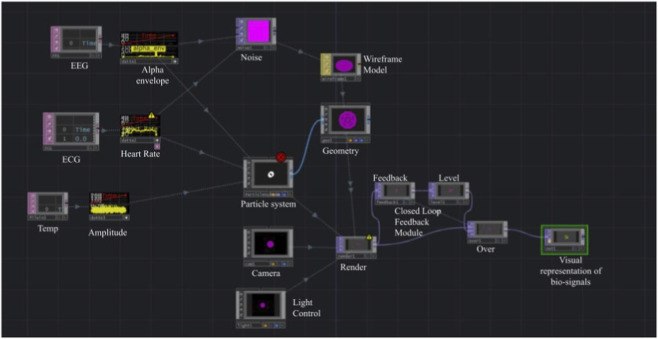
This figure illustrates the architecture of the bio-responsive multimedia environment implemented in TouchDesigner. The system integrates multimodal physiological signals–EEG (alpha envelope), ECG (heart rate), and skin temperature–into visual feedback. The processed data streams are mapped to dynamic visual parameters, modulating spatial transformations, opacity, color, and particle-based motion, thereby reflecting the user’s internal emotional and cognitive states.

The processed physiological signals were mapped to visual parameters within the immersive environment to generate bio-responsive feedback. Specifically, EEG alpha activity modulated spatial transformations within the scene, heart-rate dynamics influenced rhythmic fluctuations in visual patterns, and skin-temperature signals controlled the transparency and intensity of visual elements. The overall architecture of this bio-responsive environment is illustrated in Figure 6, where multimodal physiological signals derive dynamic visual transformations within the immersive interface.

**FIGURE 7:**
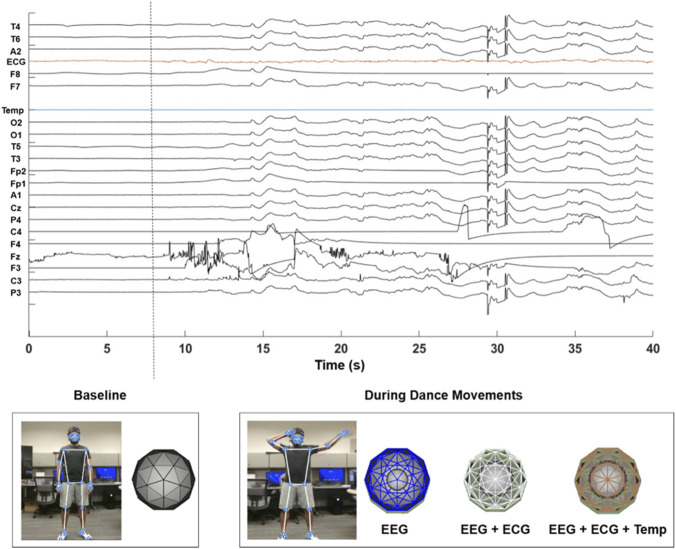
This figure illustrates the visual feedback environment driven by multimodal physiological signals. In the baseline state (left panel), the visualization remains minimalistic–displaying a grayscale image devoid of dynamic patterns–indicating a neutral physiological profile as indicated by the signals. In the dance-engaged state (right panel), physiological variability modulates distinct visual parameters: EEG alpha envelopes introduce evolving internal textures and spatial patterns, ECG-derived heart rate modulates the morphology and pulsation of the central visual node; and skin temperature dynamically adjusts the global opacity of the scene.

Through this mapping, the user’s internal physiological state continuously influences the visual characteristics of the virtual environment, establishing a feedback loop between neural activity, autonomic arousal, and sensory-motor interaction. An example of the resulting physiological-driven visualization during user interaction with the SIVAM platform is presented in Figure 7, demonstrating how variations in neural and autonomic signals dynamically alter the virtual environment. Although the current setup uses pre-recorded biosignals, the architecture supports real-time streaming enabling live biofeedback, adaptive choreography, and multi-user affective interaction in therapeutic dance scenarios.

### 3.3 Robotic mirroring and synergy-based affective inference

To achieve real-time embodied synchronization across robotic agents, the SIVAM platform employs a custom multimodal landmark integration framework that fuses MediaPipe Pose, MediaPipe Hand, and a hybrid custom pose and hand extraction model. This architecture enables the simultaneous capture of gross and fine motor gestures, producing a unified spatiotemporal representation of human movement suitable for robotic retargeting. For motion mirroring, distinct mapping strategies were applied based on each robot’s mechanical and kinematic configuration and constraints. Specifically, the MediaPipe Pose output stream was mapped to the Unitree G1, leveraging its 23-DoF full-body structure optimized for limb and torso dynamics, whereas the MediaPipe Hand and custom hand and pose fusion streams were mapped to Mitra. This rationale stems from each robot’s inherent capabilities: Mitra’s 5 DoFs per hand enable independent finger articulation and dexterous gesture reproduction, while Unitree G1’s three-finger, 7 DoFs manipulator favors coordinated gross-motor movements over isolated finger control. By distributing the control hierarchy across these complementary subsystems, SIVAM achieves fine motor gestures and expressive hand poses mirrored by Mitra, while full-body pose trajectories and rhythmic dance-based limb coordination are rendered through Unitree G1. The mapping is achieved using a nonlinear joint-to-joint correspondence model, incorporating inverse kinematics and motion-space scaling to preserve anatomical proportions and continuity across frames.

Human motor synergies reflect how the central nervous system organizes movement under different internal states. In stress or high-arousal conditions, motor control may shift, causing changes in the synergy structure such as more co-contraction, reduced variability, or altered coupling. By extracting the synergy vectors from behavioral movement such as dance or hand gestures, one can detect deviations from a user’s baseline non-stress signature. In effect, these deviations in synergy space serve as a behavioral biomarker for latent stress. Prior work in prosthetics and assistive robotics has shown that synergy-based decoding can yield robust online motion classification for upper-limb movements compared to time-domain features, even under noisy conditions (Antuvan et al., 2016). Similarly, synergy frameworks grounded in neuroscience show that the motor cortex encodes hand gestures as synergy combinations, reinforcing the interpretability of such representations (Leo et al., 2016). Moreover, in studies of finger motor control, researchers have successfully used synergy analysis to quantify fine-motor function differences in clinical populations (Sorimachi et al., 2025). When synergy features are aligned with simultaneously recorded physiological signals–such as EEG, ECG, GSR, EMG, and skin temperature–a richer multimodal model of stress *versus* non-stress emerges. Physiological indices such as decreased heart rate variability, elevated skin conductance, altered EEG power bands constitute well-validated markers of sympathetic activation and mental stress. By correlating changes in motor synergies with these physiological shifts, a classifier can more reliably distinguish between stressed and non-stressed states that either modality alone. This synergy-physiology fusion supports therapy personalization: when the system detects movement synergies drifting towards stress subspace, it can adapt to the next exercise such as slow tempo, simpler choreography, calming audio cues to reduce cognitive-arousal load. Over repeated sessions, the system can track how a user’s synergy-physiology relationship evolves–serving as an objective metric of resilience or intervention efficacy.

## 4 Discussion

The primary target users for SIVAM are individuals with mental health conditions such as anxiety, depression, and schizophrenia who may benefit from dance movement therapy but face barriers to in-person attendance (Koch et al., 2019; Wang et al., 2022; Wu et al., 2022). Secondary users include adults with subjective cognitive decline or mild cognitive impairment, and individuals with Parkinson’s disease who may benefit from structured movement therapy (Wu et al., 2021). The current study employed a focused participant pool (N = 5) to conduct an initial validation of the SIVAM platform, emphasizing system-level performance, integration fidelity, and real-time responsiveness rather than on therapeutic efficacy. This validation strategy follows procedures set by similar studies that prioritized engineering feasibility over therapeutic outcomes. For instance, MIDAS system–an immersive multisensory rehabilitation system–was evaluated in a pilot study with five subjects focusing on feasibility, motivation and safety (Kow et al., 2022). Similarly, VR-based rehabilitation prototypes like combining motion-based feedback and smart wearable sensor (Baron et al., 2023) were first validated with small cohorts to iterate on architecture and interaction mechanism before scaling up.

By adopting this iterative system-oriented methodology, the current study was able to benchmark SIVAM’s performance across domains including sensor fusion capabilities, communication latency, avatar mapping fidelity, closed-loop responsiveness and usability. The participant group was purposefully diversified to include both experienced dancers and individuals with no formal movement training, allowing us to stress-test the system across a range of motor complexity, affective engagement, and kinematic variation.

### 4.1 System performance and multimodal integration

The results demonstrate successful integration and functionality of the SIVAM platform across multiple modalities. Real-time embodied synchronization was achieved with both robotic agents, and synergy features were successfully extracted and aligned with physiological signals. This confirms that the platform can serve as a foundation for closed-loop therapeutic interventions. The integration of physiological signals with the TouchDesigner visualization framework demonstrated the potential for creating bio-responsive environments that reflect the user’s internal state. By mapping EEG alpha envelope to spatial data, heart rate to visual pulsation, and skin temperature to opacity, the system creates a continuous feedback loop between neural activity, autonomic arousal, and sensory-motor output. Although the current setup uses pre-recorded biosignals, the architecture supports real-time streaming enabling live biofeedback, adaptive choreography, and multi-user affective interaction in therapeutic dance scenarios. This capability positions SIVAM to move beyond passive sensing toward active, embodied therapeutic engagement.

The synergy-based approach to affective state inference represents a core analytical mechanism of SIVAM. By extracting movement synergies and aligning them with physiological signals, the system can detect subtle deviations in motor behavior linked to emotional dysregulation. This capability enables personalized intervention adaptation and provides an objective metric for tracking treatment response over time.

### 4.2 Limitations

Despite the successful integration and functionality across multiple modalities, several limitations were observed. The MediaPipe-based tracking system utilizes 2D pose estimation models operating on image plane projections from RGB video input. This reliance on 2D landmark detection introduces tracking instability when participants rotate their torso or limbs significantly out of the frontal camera plane. During fast-paced or rotational dance movements, instances of landmark dropout were observed, leading to transient inaccuracies in avatar pose rendering. These occlusion-related errors degrade the realism of the avatar mirroring and reduce the responsiveness of gesture-based feedback loops. The MediaPipe Hand model demonstrated reduced fidelity in tracking fine-motor gestures involving close finger proximity or occlusion. Gestures such as clasped hands or intricate finger movements resulted in ambiguous landmark prediction for few frames. In such cases, the algorithm often failed to resolve individual digit trajectories. This limitation is particularly critical in therapy applications that rely on accurate recognition of subtle finger poses for emotion expression or functional rehabilitation. The current study employed a healthy participant cohort for initial technical validation. While this was intentional to establish system robustness under ideal conditions, it limits generalizability to clinical populations pending future trials. The current core architecture is designed for compression, and the pilot study validated the core technical pipeline under ideal conditions. However, form-factor compression to standalone platforms suitable for home deployment remains an engineering challenge to be addressed in parallel with clinical translation.

## 5 Conclusion

This study presents SIVAM, a multimodal platform for delivering dance movement therapy through the integration of markerless motion capture, physiological sensing, immersive virtual environments, and humanoid robotic interaction. The key contributions are: (i) a unified architecture that synchronizes multimodal physiological signals with full-body and hand motion capture in real time, (ii) the introduction of dual interaction modes that enables both self-observation and guided therapeutic engagement, and (iii) a synergy-based framework that links movement patterns with physiological signals for interpretable affective state inference.

The initial technical validation with five participants demonstrates that the system can reliably capture and synchronize multimodal data, support real-time avatar and robot mirroring, and maintain stable performance across users with varying movement complexity. These results establish the feasibility of SIVAM as a platform for embodied, technology-mediated therapy.

While this study focuses on system-level validation, future work will involve clinical evaluation with target populations, improved robustness for complex and occluded movements, and the integration of real-time biofeedback and adaptive therapeutic protocols. The SIVAM platform provides a framework for extending embodied therapies beyond traditional clinical settings and enabling accessible, home-based health interventions.

## Data Availability

The raw data supporting the conclusions of this article will be made available by the authors, without undue reservation.
